# Supplementary material for: ASC oligomer favors caspase-1CARD domain recruitment after intracellular potassium efflux
Source: J Cell Biol. 2023 Jul 4;222(8):e202003053. doi: 10.1083/jcb.202003053 (PMC10318405; doi:10.1083/jcb.202003053)
Supplement: Table S1 — shows detection of ASC with α-PYDASC (SantaCruz) and α-CARDASC (BioLegend) antibodies. [file JCB_202003053_TableS1.docx]

**Supplemental Table S1. Detection of ASC with α-PYD_ASC_ (SantaCruz) and α-CARD_ASC_ (Biolegend) antibodies**

| **ASC specie** | **Tag** | **Antibody** | **ASC** | | |
| --- | --- | --- | --- | --- | --- |
|  |  |  | **Soluble** | **Oligomeric** | **Oligomeric with low K^+^** |
| Human | − | α-PYD_ASC_ | + | + | + |
|  |  | α-CARD_ASC_ | + | + | ++ |
| Mouse | − | α-PYD_ASC_ | + | + | + |
|  |  | α-CARD_ASC_ | − | + | ++ |
| Mouse | YFP | α-PYD_ASC_ | + | + | + |
|  |  | α-CARD_ASC_ | − | − | + |

(-) denotes no staining; (+) denotes staining; (++) denotes an increase of staining.
